# Scopolamine as an Anaesthetic

**Published:** 1906-11

**Authors:** 


### Scopolamine as an Anaesthetic.

Sharp says that a few of the advantages of scopolamine anesthesia are: The harmlessness of scopolamine—while the pulse is accelerated the quality remains good and strong; the absence of the stage of exhaustion; the small amount of chloroform used; the natural sleep after the operation, extending over the period of postoperative pains; the absence of nausea and other ill effects which usually follow the use of chloroform alone; the deep, full, and regular respiration; the ability of the patient to take water or even food shortly after awakening without nausea or vomiting. This last the author believes to be of great importance. By being able to give liquids the thirst which follows operation is relieved, and at the same time a natural vehicle to carry away waste matter is furnished.—*Therapeutic Gazette.*

